# MGERT: a pipeline to retrieve coding sequences of mobile genetic elements from genome assemblies

**DOI:** 10.1186/s13100-019-0163-6

**Published:** 2019-05-14

**Authors:** Andrei S. Guliaev, Seraphima K. Semyenova

**Affiliations:** Laboratory of Genome Organization, Institute of Gene Biology of the Russian Academy of Sciences, Vavilov Str., 34/5, Moscow, 119334 Russia

**Keywords:** Mobile genetic elements, Retrotransposons, Genome analysis, Schistosoma, Flatworms, Penelope, PLE

## Abstract

**Background:**

Genomes of eukaryotes are inhabited by myriads of mobile genetic elements (MGEs) – transposons and retrotransposons - which play a great role in genome plasticity and evolution. A lot of computational tools were developed to annotate them either in genomic assemblies or raw reads using de novo or homology-based approaches. But there has been no pipeline enabling users to get coding and flanking sequences of MGEs suitable for a downstream analysis from genome assemblies.

**Results:**

We developed a new pipeline, MGERT (Mobile Genetic Elements Retrieving Tool), that automates all the steps necessary to obtain protein-coding sequences of mobile genetic elements from genomic assemblies even if no previous knowledge on MGE content of a particular genome is available.

**Conclusions:**

Using MGERT, researchers can easily find MGEs, their coding and flanking sequences in the genome of interest. Thus, this pipeline helps researchers to focus on the biological analysis of MGEs rather than excessive scripting and pipelining.

**Electronic supplementary material:**

The online version of this article (10.1186/s13100-019-0163-6) contains supplementary material, which is available to authorized users.

## Background

Eukaryotic genomes are mostly repetitive by nature. Mobile genetic elements (MGEs), discovered by B. McClintock [1], constitute a major part of the repetitive fraction. Usually, MGEs are divided into two broad classes: Class I elements, or retrotransposons known to mobilize themselves through RNA intermediate and “copy-and-paste” mechanism; and Class II elements, or DNA transposons mostly mobilizing through “cut-and-paste” mechanism [2–4]. It is commonly accepted that retrotransposons or retroelements are represented by four classes (or orders): elements with long terminal repeats - LTR-retrotransposons, without terminal repeats - non-LTR-retrotransposons (including non-autonomous SINEs), *Penelope*-like elements (PLEs) and Tyrosine-recombinase retrotransposons (DIRS) [3]. Each class/order includes several superfamilies, e.g. *L1*, *R2*, *R4*, *RTE*, *CR1*, CRE, *Jockey*, *Tx1, Rex, I, RandI, NeSL* in non-LTR [2, 5], *Penelope*/*Poseidon*, *Perere*, *Neptune*, *Coprina, Athena* in PLE [6], *DIRS, Ngaro* and *VIPER* in DIRS [3]*.* Although this system is a subject of debate [7] and new discoveries like giant *Terminons* elements [8] may result in revising of the existing scheme, we will refer to this classification throughout this paper. Nowadays it is widely accepted that MGEs and particularly retrotransposons play a great role in genome plasticity and evolution [9–14]. Besides transposition machinery, they often encode diverse regulatory motifs (promoters, enhancers, regulatory RNAs) [15–17] and ribozymes [18] and may serve as recombination sites causing chromosomal rearrangements and structural variations as well as participate in creation of new genes and destruction of existing ones, and horizontal gene transfer [15, 19–23]. Modern hypotheses postulate that proliferation of MGEs in a genome occurs in response to drastic changes in environmental conditions thereby playing a role in speciation and adaptation [24, 25]. Additionally, it was demonstrated that abundances of genomic repeats contain phylogenetic signal [26].

But, despite MGEs importance, not so many researchers pay attention to the repetitive fraction [27] and this is - at least partially - caused by the lack of well-established tools/bioinformatic protocols for MGE search and analysis. Even eight years ago there was a “dense forest” of bioinformatics software [28] aimed to search for the repetitive fraction in genomic assemblies or raw reads, since then their amount increased that reflects constantly growing demands of the scientific community. All such tools could be divided into two categories according to the implemented approach: search by homology or de novo search. The software of the first type require a predefined library of repeats which is unavailable for most of the genomes. The typical and most widely used representative of this category is RepeatMasker [29], although it was designed to annotate and mask repetitive regions before the start of gene discovery. The other one - CENSOR [30] is used by RepBase - a curated database of repeats [31–33]. De novo approaches - as it could be inferred from its name - requires no previous knowledge of repeat content of the genome. Methods of this group are either structure based (i.e. use of specific structures like long terminal repeats of LTR-retrotransposons or TIRs and TSDs of MITE transposons), like LTRharvest [34], LTR_STRUC [35], LTRtype [36], MITE-Digger [37] and MITE-Hunter [38] or genome self-alignment based. This last group includes numerous tools, so we mention a few: RepeatGluer [39], RepeatFinder [40], PILER [41], RECON [42], RepeatScout [43], MGEScan [44], CARP [45], TEdenovo [46], Red [47]. PILER, RECON, RepeatGluer, RepeatFinder and RepeatScout were compared in [48] and it was shown that the best ones are RECON and RepeatScout, so their combination implemented in the package RepeatModeler [49] is still the most popular de novo repeat annotation package. Recent and more powerful tools (TEdenovo, as part of the REPET package, CARP and Red) unfortunately have their own shortcomings. The first one requires the installation of a massive set of packages on a distributed computer cluster which is not an easy task as well as a great amount of computational power required to perform the analysis. The second is not a tool per se right now, rather a collection of various programmes and libraries with a list of instructions attached. The latter of the three despite its speed and novel approach (machine learning algorithms) provides merely a list of repeats’ coordinates without any classification. Also, it is worth to mention a group of tools that use raw reads (either short or long) for de novo repeat discovery, thus being independent of assembly quality. These are RepLong, RepeatExplorer, ReAS, RepARK [50–53], but to date, their capabilities remain limited [54].

Thus, to obtain MGE’s sequences ready for phylogenetic analysis researchers have to be capable of using scripting languages and making pipelines manually to send an output of de novo programs to homology-based tools, validating found hits and retrieving coding sequences. That’s why we designed and developed a program called MGERT (Mobile Genetic Elements Retrieving Tool). Our program is actually a wrapper script that unifies several tools, hides technical quirks from a user and outputs ready-to-analyze individual copies of MGEs (either intact or disrupted) along with their flanking regions, genomic coordinates and descriptive statistics as well.

### Implementation

**Fig. 1:**
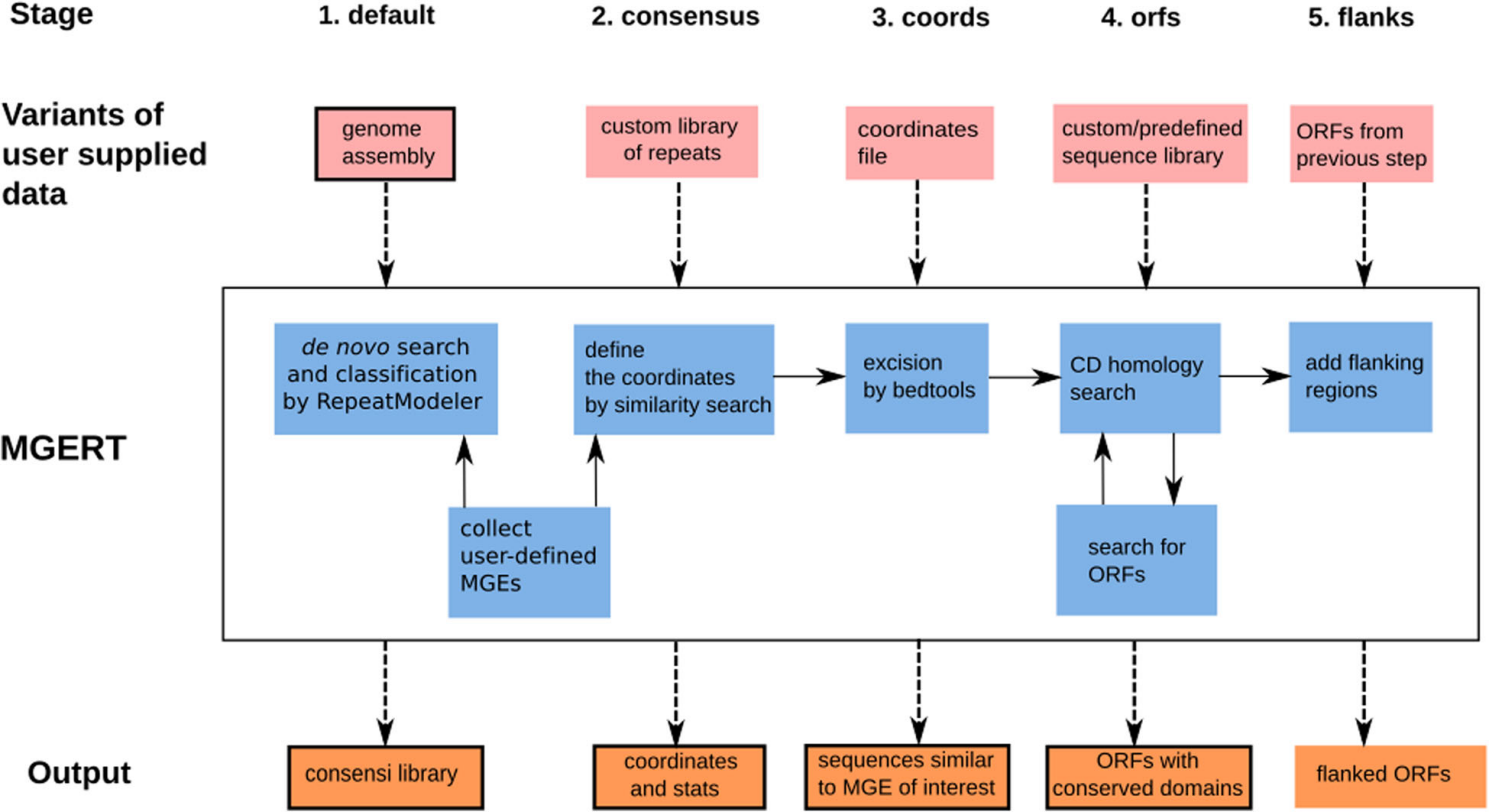
MGERT workflow chart. The genome assembly used as input for RepeatModeler for de novo search and classification of MGEs; their families that have been chosen by a user are pipelined to RepeatMasker for similarity search; these regions of similarity then being excised by BEDTools; conserved domain homology search by RPS-Blast followed by ORF extraction by ORFinder; adding flanking regions of specified length. Black outline of the input squares designates mandatory files; a black outline of output squares designates the output is used in the following step of the pipeline

The basic plan of MGERT processing is as follows: a) de novo repeat annotation in a genome assembly using RepeatModeler; b) collecting consensuses of repeats specified by user; c) search for instances of these repeats in the assembly using RepeatMasker; d) retrieving of individual copies from the assembly using BEDTools [55]; r) search for ORFs with conserved domains using RPS-BLAST and CDD [56, 57]. One may start the pipeline from any of these steps (Fig. 1).

First of all, one needs to set up the pipeline. It should be done in two consecutive steps. The first step: run the pipeline with the ‘*--configure*’ flag, what will result in a configuration file (in JSON format) where MGERT stores all the paths to executables, input and output files; if all the required tools are installed MGERT will find them automatically, else it will prompt user to enter valid paths manually. The second step: run the pipeline with ‘*--make-cdd*’ flag which will tell MGERT to prepare local RPS-BLAST database. To successfully complete this step the following conditions have to be satisfied: all the PSSM files (in SMP format) corresponding to particular domains of interest (i.e. RT or/and EN) have to be inside the working directory as well as comma- or TAB-separated table (with CSV extension) with domain - PSSM correspondence (see Additional file 1). This last table is needed to explicitly show MGERT which types of conserved domains should be validated in every single ORF. After this step new directory, “LocalCDD*”* will be created with all the necessary files inside, and the path to this directory will be added to the configuration file. After these preparation steps one can run MGERT specifying just two arguments: MGE type of interest (like LINE, PLE, BovB etc.) and name of the genome assembly (note, that MGE type should be specified according to names of the records in the FASTA file which might be checked using ‘*--check-types*’ flag). The pipeline will create a directory named after this assembly and all the output files will be saved into it.

MGERT pipeline uses the following algorithm and tools to collect user selected MGEs:

1. run the RepeatModeler pipeline and collecting MGE consensuses specified by the user (depends on RepeatModeler classification of de novo consensuses);
2. define the coordinates of regions similar to consensus sequences (by RepeatMasker);
3. merge of closely located regions in one single region; extraction of these regions (matches) to a FASTA file (using BEDTools), if the regions are located on minus strand, reverse-complement them;
4. search for conserved domains in the excised matches (by RPS-BLAST) with subsequent search for open reading frames containing CD-hit within (by both ORFinder and RPS-BLAST). By default ORFfinder uses standard genetic code table and reports ORFs of minimum length 1000 bp starting from ATG codon on a plus strand. Any of these parameters could be easily set using corresponding optional arguments;
5. add flanking regions of a specified length (default length is 0, i.e. add no flanks) (see Fig. 1)

All these steps are automated by single python script *MGERT.py.* Thus, running a single script user can easily obtain genomic sequences derived from the chosen type of MGEs as well as interrupted and intact coding sequences of MGEs of interest.

MGERT takes as input either genome assembly only or genome asembly and one of the following five files: a) custom library of repeats (or consensuses) to find in an assembly; b) coordinates file, either in RepeatMasker or bed format; c) FASTA file of excised consensus’ matches; d) file with MGE’s open reading frames to filter it out or to add flanking regions.

These five input files might be used in five corresponding modes (specifying by the flag ‘*--from-stage’*):

1. *default* mode: a user has only the genome assembly and starts from de novo search of MGEs of interest (with all the following steps);
2. *consensus* mode: a user already has de novo library of all MGEs and wants to run the remaining steps of the pipeline or to chose other MGE family for analysis;
3. *coordinates* mode: a user already has a coordinates table of MGE’s similarity regions and wants to (re) run the analysis with different excision settings;
4. *orfs* mode: a user already has excised MGEs matches and wants to (re) run the ORFs and CD search with different settings;
5. *flanks* mode: run to add flanking regions of another length to the ORFs.

Besides the standard output files of underlying tools MGERT produces the following files:

- MGE consensuses of interest (MGE_consensi.fa);
- unclassified consensuses to be sent to the CENSOR web site (Unknown_consensi.fa.classified);
- BED file with coordinates of matches (genome.out.bed);
- FASTA file of merged and excised matches (MGE_excised_matchesX.fa, where X stands for value of --*merge* option);
- Files with descriptive statistics and histograms of lengths of all output sequence datasets (Additional files 2, 3 and 4) (.stats and .png files);
- FASTA file of matches with CD hits (MGE_matches_with_hits_eX.fa, where X stands for e-value);
- FASTA file with coding sequences of a specified length (MGE_cdsX.fa, where X stands for CDS minimum length);
- FASTA file with coding sequences containing conserved domains (MGE_cdsX_with_domains_eX.fa, where X stands for CDS minimum length and e-value, respectively);
- FASTA file with the same sequences but with flanks added (MGE_cdsX_with_domains_eX_extended_LXRX.fa, where X stands for CDS minimum length, e-value, length of left (L) and right (R) flanking regions, respectively).

One can start the analysis from any of the steps described above, this is regulated by the use of the option ‘*--from-stage’* with one of the four keywords (*consensus*, *coords*, *orfs* or *flanks*) corresponding to the analysis steps from 2nd to 5th. In a case when a researcher already has a library of MGE consensus (possibly obtained from another de novo repeat prediction tool) or simply run MGERT one more time to retrieve another family of repeats, one should use the option ‘*--from-stage consensus’* and (optionally) specify the consensus file after the option ‘*--lib’*. One should run the pipeline from stage 3 (option ‘*--from-stage coords’*) if one wants to change the minimum distance between matches to be merged into a single entry (it is regulated with the ‘*--merge’* option). Running the pipeline from steps 4 and 5 (keywords *orfs* and *flanks*) can be done to change the minimum length of an ORF (along with the genetic code, start codon, strand and expectation value) and flanking regions correspondingly. Also, it is possible to specify at which step the pipeline should stop using an option ‘--*to-stage’.* It can be useful e.g. when one wants to run RepeatModeler only and inspect the results then.

Also, the pipeline allows a researcher to use CENSOR classification results to increase the yield of the MGEs of interest. The RepeatModeler outputs many unclassified consensuses (marked as ‘unknown’ in FASTA header) which one may send to the web-version of CENSOR - a repeat classification and masking tool developed and hosted (along with RepBase database) by the GIRI Institute. In such a case a researcher has to use the option ‘*--censor’* providing either URL or path to HTML file with classification results to be parsed by MGERT.

## Results and discussion

To test the pipeline we searched for *Penelope*-like elements (PLE) and non-LTR-retrotransposons of superfamily L1 in two genome assemblies: the parasitic flatworm *Schistosoma mansoni* (RefSeq accession GCF_000237925.1) and the fish *Takifugu rubripes* (RefSeq accession GCF_000180615.1), because both species have small genomes (364.5 Mb and 391.5 Mb correspondingly, i.e. it’s easier to process them), high diversity of MGE families and high-quality of assembly (assembled chromosomes) [58–61]. Typical PLE has a single ORF (ca. 2000 bp) encoding a protein with RT and EN (GIY) domains [62], while a active L1 retroelement has two ORFs: ORF1 encoding for a protein of unknown function and ORF2 (ca. 2500 bp) encoding for a protein with RT and EN activities. In our test runs we searched for ORF2 of L1, because it encodes for a RT that is suitable for phylogenetic analysis.

**Fig. 2:**
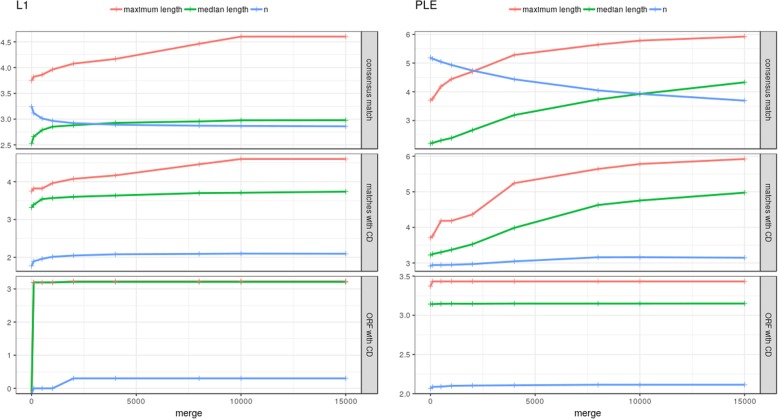
Number, median length and maximum length of PLEs and L1 hits retrieved by MGERT with different *merge* parameter

Firstly, we ran MGERT with different settings that can affect the yield of MGEs to detect optimal values of some important parameters (PSSM files used for constructing local conserved domain databases are listed in Additional file 5). MGERT has three parameters affecting the number and/or size of resulting intact MGE. These parameters are the following: “merge” to join closely located regions, “e-value” - the conservative domain (CD) hit threshold and “minimum length” of an ORF to be considered. While “e-value” used in RPS-Blast is commonly accepted to be less than or equal to 0.01 and minimum length of ORF is dependent on researcher’s goals and type of MGE, the “merge” value will result in significantly different numbers of found MGEs and seems to be arbitrary. That’s why we performed several testing runs with different values of the *merge* parameter – the greater the value is, the more MGE matches will be joined into one record. In search of optimal value we tested nine: 0, 100, 500, 1000, 2000, 4000, 8000, 10,000 and 15,000 bp (see Additional file 6). As we expected, the greater the *merge* parameter was, a) the smaller the number of hits was; b) the greater the median length of hits was; c) the longer the maximum length of hits was. The optimal value of the *merge* parameter is expected to be no greater than the full-length of a MGE’s ORF because it is very unlikely that such a big gap will be missed or fragmented by RepeatMasker. We found that starting from *merge* = 2000 bp for both types of MGEs all the statistics reached the plateau (Fig. 2), so we considered it as an optimal value and used it in the following analysis. To generalize, in order to find an optimal value of *merge,* we can suggest users either to run the pipeline several times with different *merge* values or to set *merge* to the expected length of MGE/ORF of interest.

With such settings we found in the genome of *T.rubripes* just 2 ORFs encoding both RT and EN domains and starting from ATG-codon (1686 bp and 1566 bp in length) and 7 ORFs encoding both RT and EN domains but starting from any sense-codon with the median length of 1980 bp (minimum length 1569 bp, maximum length 2220 bp, Additional file 11). Apparently, all L1 copies that we found are inactive due to the lack of initiation codon or small size of putative ORF. More interesting fact is that original RepeatMasker annotation provides no data on L1 in the *T.rubripes* genome. However, using MGERT we found 9 consensuses related to L1 which contributed to 0.24% of the *T.rubripes* genome. This is in contrast with the absence of L1 elements in the current MGE annotation of the *T.rubripes* genome, and suggests that a consensus for a L1 element in RepBase is missing. The closest homologous sequence that can be found in RepBase is called «KibiFr1 non-LTR retrotransposon» [63] and it is impossibe to link it with L1 superfamily.

**Fig. 3:**
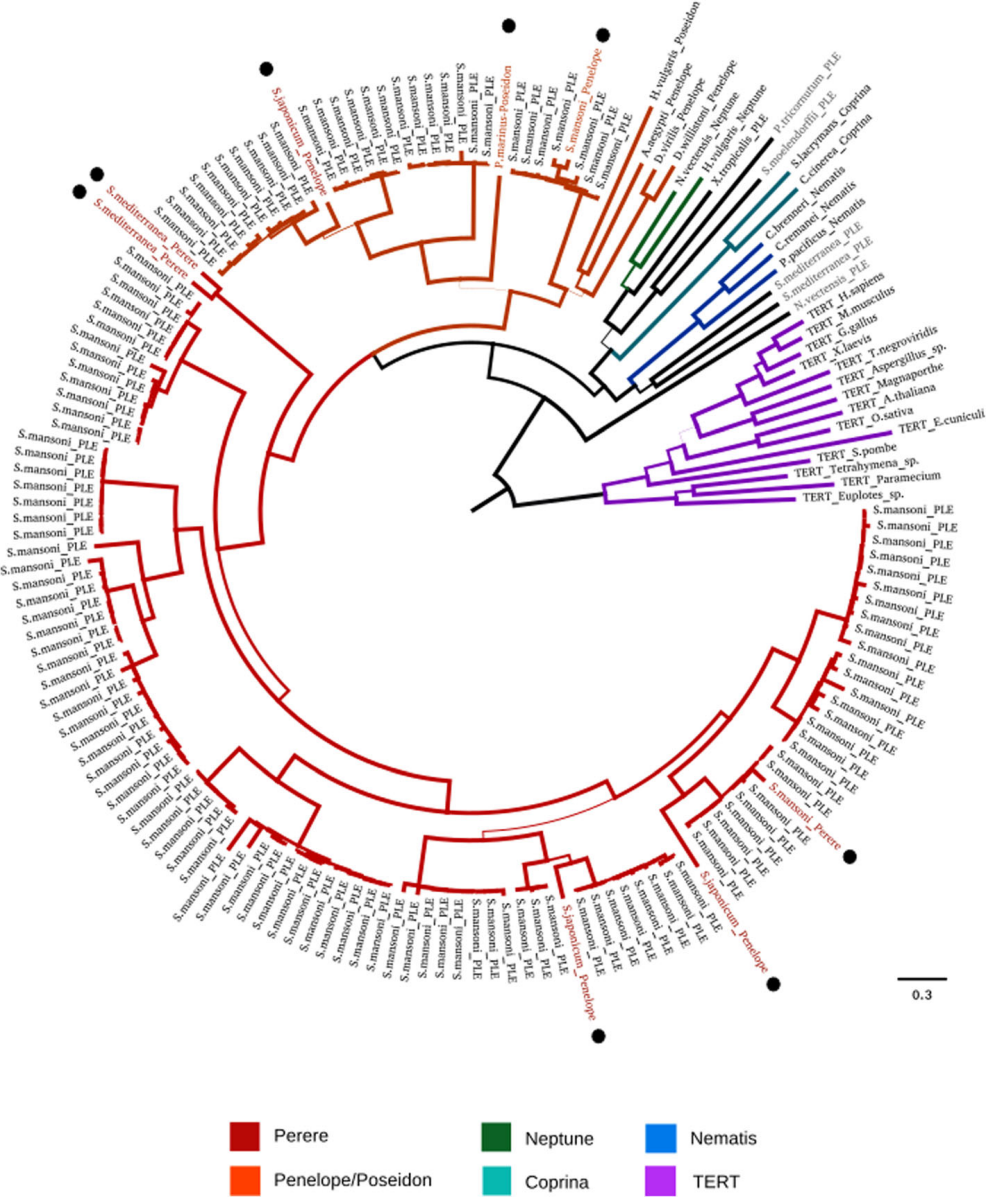
Phylogenetic tree based on amino acid sequences of PLEs found in the genome of *S.mansoni* by MGERT and PLE representatives from RepBase. TERT sequences were used as the outgroup. Blacktip labels represent PLE found by MGERT, other colours - PLE obtained from RepBase. Black circles designate previously known PLEs from genus *Schistosoma.* Tree branch width corresponds to bootstrap support value (less or greater than 50)

Even more interesting things were revealed when we searched for PLEs. With the same settings, we found a remarkable diversity of PLEs in the genome of *S.mansoni* - 167 ORFs encoding both RT and EN (GIY-YIG) domains with the median length of 1387 bp (minimum length set to 1000 bp, maximum length 2706 bp). To assess whether found PLEs are related to known PLE families we performed a phylogenetic analysis of translated protein sequences as follows: coding sequences were aligned using MAFFT v7.271 [64] (alignment strategy *--auto*), the resulting alignment was trimmed by trimAl v1.2 [65] (with *--gappyout* option) and the phylogenetic tree was built using RAxML v8.2.12 [66] and visualised by FigTree v1.4.2 [67], best-fit substitution model was selected using ModelTest-NG v0.1.15 [68]. As reference sequences we used 27 PLEs from RepBase belonging to different families and different host organisms (including five ones from Schistosoma flatworms, see Additional file 7) and 14 sequences of telomerase reverse transcriptases (TERT, the dataset from [69] as an outgroup (see Fig. 3 and Additional files 8, 9, 10, 11). We found that all newly discovered PLEs of the trematode worm belong to two families: *Perere* and *Penelope/Poseidon* and that they form clusters with Schistosoma-specific PLEs from RepBase as well. Interestingly, *Penelope/Poseidon* family includes two subfamilies diverged as long ago as the common ancestor of Chordates and Invertebrates existed. The second (and the most numerous) part of *S.mansoni* PLEs forms a diverse group of *Perere* family sharing common ancestry with PLEs from free-living flatworm *Schmidtea mediterranea*. At least one clade from this group is common for *Schistosoma* genus because it includes PLEs from sister species *S.japonicum*.

Finally, we compared genome percentage occupied by PLEs according to original repeat annotation and the one made by MGERT. We extracted all records related to PLEs from the RepeatMasker output table available through NCBI as part of the original genome annotation report. We found that their summary length makes up 0.84% of the whole genome. On the other hand, consensus library of PLEs made by RepeatModeler (as part of the MGERT pipeline) allowed us to mask 9.13% of the *S.mansoni* genome, of them matches encoding both RT and EN (GIY-YIG) domains occupy about 1.11% of the genome, while intact ORFs (i.e. ORFs of any length containing both PLE-specific domains - the settings used are provided below) occupy about 0.058% of the genome (lower boundary of PLE abundance estimates). Such a great difference in PLEs abundance estimates, was caused by dramatically incomplete repeat library provided by GIRI/RepBase that was used for original repeat annotation. So, besides some critical notes on RepeatModeler performance [54], one should not avoid its use for generating de novo repeat libraries in favour of homology-based repeat annotation only.

These results clearly demonstrate how effective the MGERT pipeline could be for the investigation of the abundance, diversity and phylogenetic relationships of retroelements within major superfamilies, and apart from this how understudied *Penelope-*retrotransposons are.

## Conclusions

Instead of extensive coding and manipulating with outputs of several tools, researchers using MGERT can easily obtain protein-coding sequences of mobile genetic elements of interest from genomic assemblies and use them for further analysis even if no previous knowledge on MGE content of a particular genome is available. The pipeline potentially can be used with output of any existing de novo repeat search and classification tool.

## Availability and requirements

Project name: MGERT.

Project homepage: https://github.com/andrewgull/MGERT

Operating systems: Linux.

Programming language: Python 3.

Other requirements: RepeatModeler, RepeatMasker, BEDTools, ORFinder, RPS-Blast.

Licence: GNU GPL v3.

Any requirements to use by non-academics: none.

## Additional files

Additional file 1: **Table S1.** PSSM-domain correspondence file example (XLSX 3 kb)

Additional file 2: **Table S2.** Descriptive statistics of lengths of found PLE hits calculated by MGERT (XLSX 4 kb)

Additional file 3 **Figure S1.** Histograms of PLE hits lengths distribution produced by MGERT (PNG 87 kb)

Additional file 4: **Figure S2**. Histograms of L1 hits lengths distribution produced by MGERT (PNG 82 kb)

Additional file 5: **Table S3.** PSSM files used for local CDD construction (XLSX 6 kb)

Additional file 6: **Table S4.** Results of MGERT testing runs (XLSX 10 kb)

Additional file 7: **Table S5.** Reference PLEs used for phylogenetic analysis (XLSX 4 kb)

Additional file 8: Penelope_cds1000_RTEN_e01.fa Coding sequences of PLEsretrieved by MGERT and used in phylogenetic analysis (FA 209 kb)

Additional file 9: ple + ref + tert.faln.trim.faa.uniq.faa Aligned and trimmed sequence dataset used for phylogenetic tree construction. (FAA 72 kb)

Additional file 10: TERT.faa Telomerase RT sequence data from Arkhipova, 2006 (FAA 8 kb)

Additional file 11: L1_cds1000_with_RTEN_e01.fa Coding sequences of L1 retrieved by MGERT (FA 13 kb)

## Abbreviations

CD: Conserved domain
CDD: Conserved domain database
EN: Endonuclease
MGE: Mobile genetic elements
ORF: Open reading frame
PLE: Penelope-like element
PSSM: Position-specific scoring matrix
RT: Reverse transcriptase
