# Supplementary figures and images for: MGERT: a pipeline to retrieve coding sequences of mobile genetic elements from genome assemblies
Source: Mob DNA. 2019 May 14;10:21. doi: 10.1186/s13100-019-0163-6 (PMC6515669; doi:10.1186/s13100-019-0163-6)

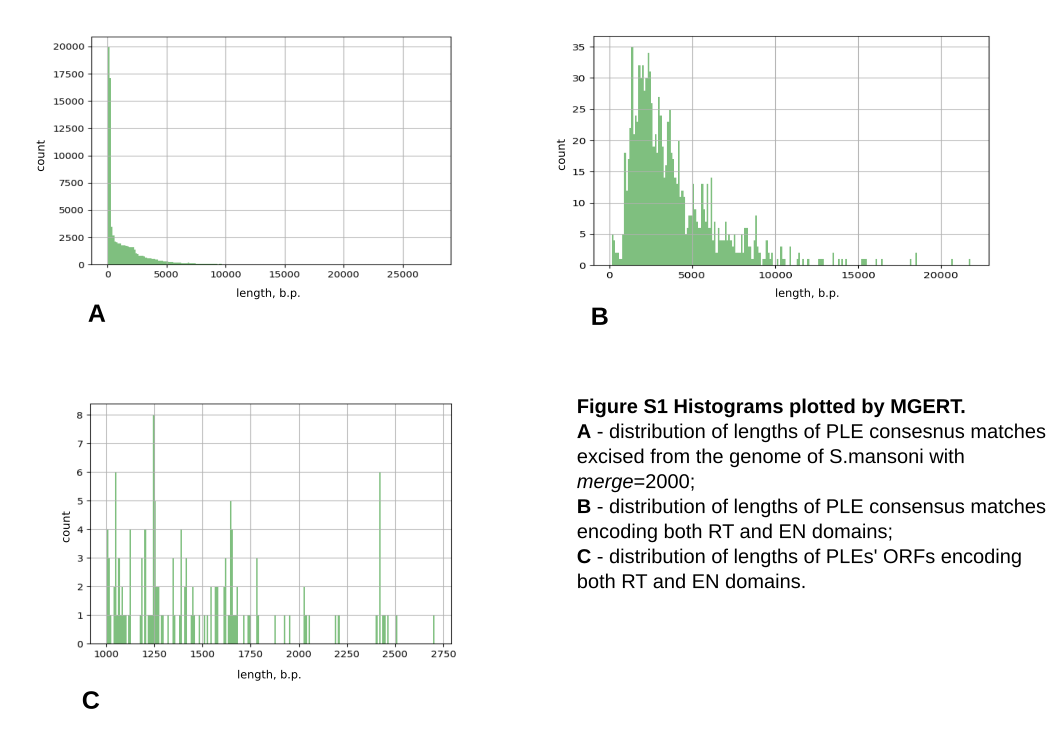

Supplement: Supplementary file 3 — Figure S1. Histograms of PLE hits lengths distribution produced by MGERT (PNG 87 kb) [file 13100_2019_163_MOESM3_ESM.png]

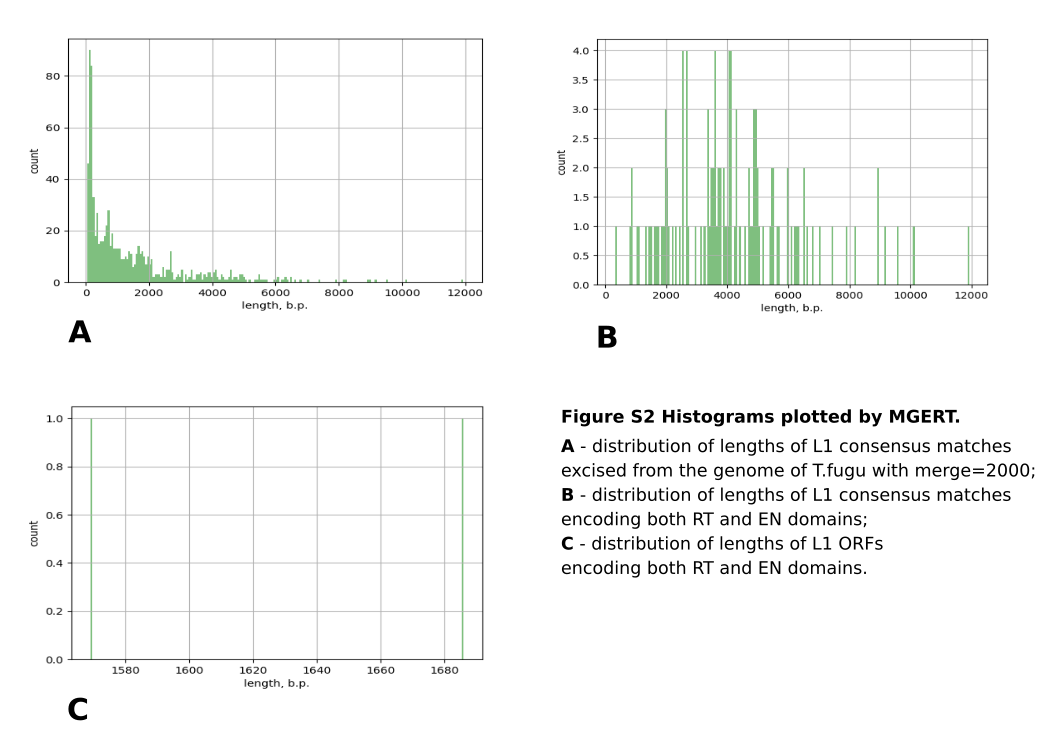

Supplement: Supplementary file 4 — Figure S2. Histograms of L1 hits lengths distribution produced by MGERT (PNG 82 kb) [file 13100_2019_163_MOESM4_ESM.png]
